# Successful treatment of an EBV‐positive HIV‐associated polymorphic B‐cell lymphoproliferative disorder by rituximab monotherapy

**DOI:** 10.1002/jha2.247

**Published:** 2021-06-05

**Authors:** Mohamad Sabbah, Sylvain Choquet, Agathe Maillon, Clotilde Bravetti, Marine Baron, Frédéric Charlotte, Damien Roos‐Weil

**Affiliations:** ^1^ Sorbonne Université Service d'Hématologie Clinique Hôpital Pitié‐Salpêtrière Paris France; ^2^ Sorbonne Université Service d'Hématologie Biologique Hôpital Pitié‐Salpêtrière Paris France; ^3^ Sorbonne Université Service d'Anatomo‐Pathologie Hôpital Pitié‐Salpêtrière Paris France

**Keywords:** HIV, polymorphic B‐cell lymphoproliferative disorder, rituximab

Dear Editor,

The incidence of lymphoproliferative disorders (LPDs) is significantly increased in immunocompromised populations, including human immunodeficiency virus (HIV)‐infected patients and solid organ or hematopoietic stem cell transplantation recipients. The majority of HIV‐related lymphomas are clinically aggressive, high‐grade monoclonal B‐cell lymphomas. In contrast, post‐transplantation lymphoproliferative disorders (PTLD) represent a heterogeneous group of lesions as PTLD consisted in either polymorphic or monomorphic proliferation that could be polyclonal, oligoclonal or monoclonal, while the vast majority is of B‐cell origin and driven in half of the cases by Epstein‐Barr virus (EBV) [1].

EBV‐associated polymorphic B‐cell LPD resembling polymorphic PTLD is a rare but recognized complication of HIV [2]. However, less than 20 cases have been published in the literature, and data regarding their treatment and outcomes are extremely scarce.

We describe here a 36‐year‐old male HIV‐infected patient treated for more than 10 years by highly active antiretroviral therapy (HAART) with sustained viremic control and no history of opportunistic infection. He was hospitalized in our institution for acute and invalidating abdominal pain associated with rectal tenesmus that did not improved despite symptomatic treatment. Routine biological analyses did not show any abnormality with the exception of polyclonal hypergammaglobulinemia (35 g/L). CD4+ T‐cell count was 396/μl (normal range, 692–1939), and HIV viral load was negative. Plasma EBV quantitative PCR was positive at 14,769 copies/ml (4.1 log). Cervico‐thoraco‐abdomino‐pelvic computed tomography‐scan revealed a circumferential thickening of the lower and middle rectum associated with multiple lymph nodes of the mesorectum of 5 mm each. No other abnormality was objectified. Recto‐sigmoidoscopy identified a large budding and ulcerative lesion extending from the lower rectum to 10 cm above. All infectious tests were negative.

Histopathological examination of the rectal mass biopsy showed a diffuse and polymorphic infiltration of the submucosae by B‐cells and polytypic plasma cells (Figure 1A). The size of CD20‐positive B‐cells was highly variable and staining for Ki67 heterogeneous. Epstein‐Barr encoding region in situ hybridization was positive in large B‐cells. CMV, HHV8, and IgG4 staining were negative. Interestingly, B‐cell clonality detection carried out by PCR analysis of immunoglobulin heavy and light chain gene rearrangement, performed on this biopsy, did not identify any monoclonal B‐cell population (Figure 1B).

**FIGURE 1:**
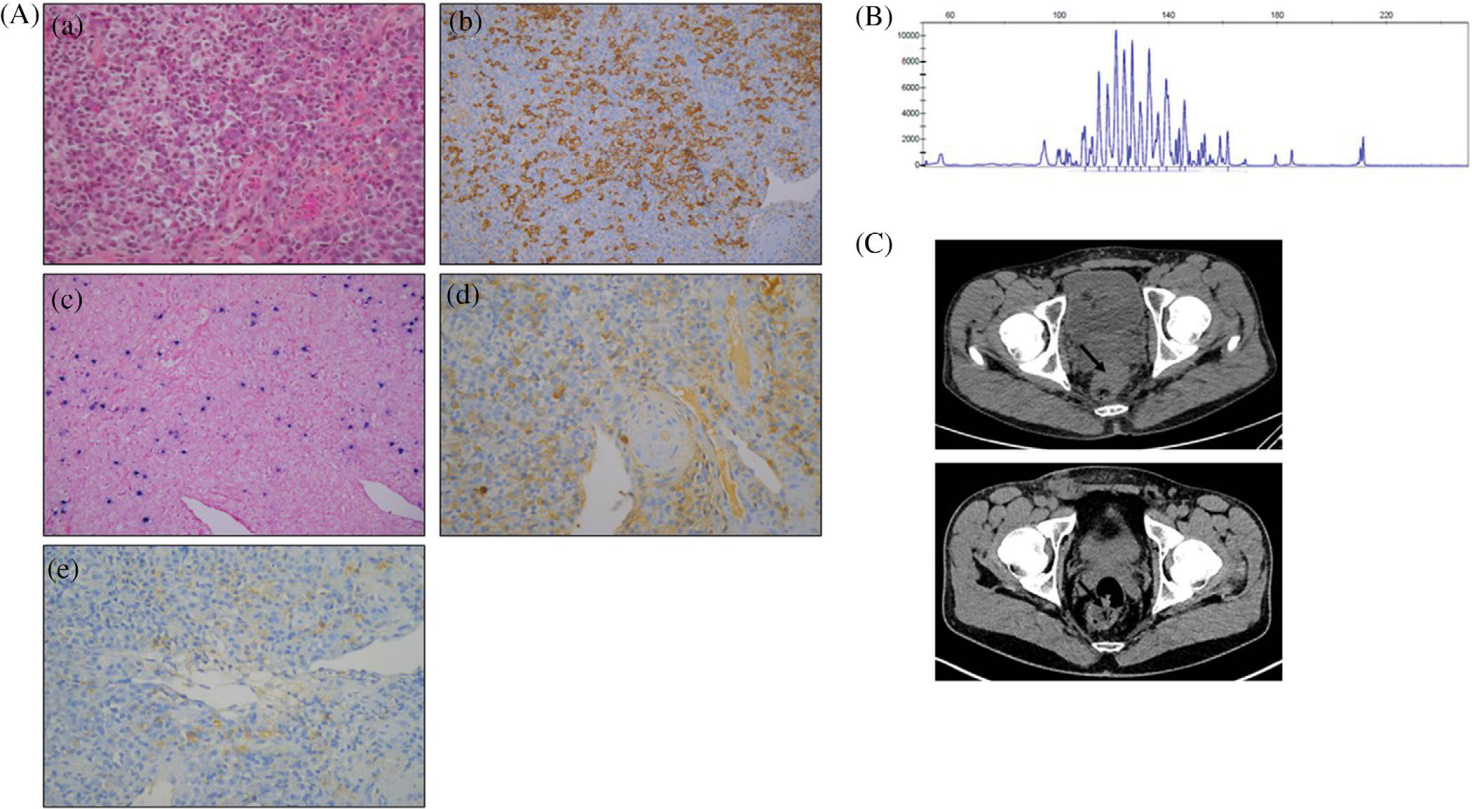
(A) Rectal mass biopsy showed (a) polymorphous infiltrate made up of variable size lymphocytes mixed with plasma cells (H&E staining), (b) CD20‐positive lymphocytes of variable size (CD20 immunostaining), (c) few EBV‐positive cells (EBV in situ hybridization, EBER probe), and polytypic expression of light chain immunoglobulin by plasma cells ((d) kappa and (e) lambda light chain immunostaining). (B) B‐cell clonality profile (Genemapper software): polyclonal aspect at the locus of immunoglobulin heavy chain gene. (C) Computed tomography (CT)‐scan images of rectal mass (indicated by black arrow) before (up) and after (down) rituximab monotherapy. No PET‐scan was available for this patient due to the lack of social security

Histological aspect and association with EBV were reminiscent of polymorphic PTLD and led to the diagnosis of HIV‐associated polymorphic B‐cell LPD. Restoring immunity was not applicable in our patient given the good immuno‐virological control obtained under HAART. We thus decided to use the same strategy as described in PTLD [3] starting with rituximab monotherapy. The patient received intravenous infusions of rituximab (375 mg/m^2^), once weekly for 4 weeks, leading to complete clinical and radiological (Figure 1C) responses and clearance of plasma EBV PCR. Four additional rituximab infusions were performed every 3 weeks. The patient did not receive any additional medication including no anti‐infectious prophylaxis. Tolerance was excellent without any infectious complication. At the end of this therapeutic sequence and 6 months later, complete clinical and radiological responses persisted, and plasma EBV PCR was still negative.

To our knowledge, this case is the first report of HIV‐associated polymorphic B‐cell LPD treated by rituximab monotherapy. HIV‐associated polymorphic B‐cell LPDs share many features with polymorphic PTLD including characteristic polymorphic B‐cell composition along with a variable plasmacytic differentiation, potential pathogenic role of EBV, and lack of additional genetic alterations. Most reported cases contained a clonal B‐cell population but usually in minority [2]. Interestingly, the B‐cell population was polyclonal in our case, which could represent an early step of LPD development before emergence of obvious monoclonality through the occurrence of additional genetic events as *MYC*, *BCL6*, and/or *TP53* abnormalities.

A potential differential diagnosis that has been discussed in our case report was an EBV‐positive mucocutaneous ulcer (EBVMCU). EBVMCU is a specific type of immunodeficiency‐associated LPD that may arise in HIV setting and share overlapping features with HIV‐associated polymorphic B‐cell LPD as mucosal involvement and favorable evolution without chemotherapy. We ruled out this hypothesis because EBVMCU presents macroscopically primarily as an ulcer, whereas our case presented more like a budding tumor with some ulcerated areas. More, histological examination did not reveal any necrosis, while it is a usual feature of EBVMCU.

Finally, very few data are available in the literature regarding treatment of HIV‐associated polymorphic B‐cell LPD. Polychemotherapy including R‐CHOP [2, 4] and restoring immunity by introducing HAART [4, 5] have been reported but in few cases and with variable success. Although longer follow‐up and additional cases are mandatory, rituximab monotherapy could represent, as described for PTLD, a safe and efficient therapeutic option in this rare entity.

## CONFLICT OF INTEREST

The authors declare that there is no conflict of interest that could be perceived as prejudicing the impartiality of the research reported.

## AUTHOR CONTRIBUTIONS

Mohamad Sabbah, Sylvain Choquet, Agathe Maillon, Clotilde Bravetti, Marine Baron, Frédéric Charlotte, and Damien Roos‐Weil performed, designed the research study, analyzed the data, and wrote the paper.
